# On Functional Medicine

**Published:** 1872-07

**Authors:** Willoughby F. Wade

**Affiliations:** Physician to the General Hospital, formerly Professor of the Practice of Physic in the Queen’s College, Birmingham


### SELECTIONS.

### ON FUNCTIONAL MEDICINE.

By Dr. Willoughby F. Wade, B. A., Physician to the General Hospital, formerly Professor of the Practice of Physic in the Queen's College, Birmingham.

In practical medicine, as in morals, it is well to have a lofty ideal. If we aim at a high mark we shall shoot higher than if we aimed at a low one. But in both there is danger in having too lofty an ideal. We feel so utterly unable to reach that which we profess to ourselves we ought to reach, that we do not long continue to aim at it. This is the case with many young practitioners of medicine. They set out impressed with the advantages of minute investigation, of active research, and of exhaustive diagnosis by those various means and instruments which they see used in hospitals. They find afterwards, when pressed by the cares and fatigue of a large practice, that they are almost compelled to forego their use; they find also that in many cases they get on pretty well without them, and thus fall into the habit of neglecting them, even in those cases where their use is, for purely practical reasons, imperative. They thus in course of time dissociate themselves from scientific medicine, which they come at last to despise. That this conclusion is actually arrived at by a large number of practitioners, many of whom do not hesitate to avow it, I appeal to the experience of every one; and I think I am not wrong in the genesis to which I have attributed it. I have long been convinced that this state of things cannot be rectified by the earnest and genuine exhortations of teachers. If it could have been, it would never have existed. It is the result of too high an ideal and the inexorable logic of circumstances.

The ideas or principles which may, I think, eventually lead to an alteration of this unsatisfactory condition may be summed up in the term "Functional Medicine." In explanation of this term, and of the ideas involved in it, I request your attention to the following considerations. What I mean by it is this: that whenever we come to treat a case, to prescribe drugs or particular diets, rest or action, we should first of all consider what function of the body it is that is improperly performed. To the setting right of that function we should address ourselves. It may be, and indeed generally is the case that more than one function is (it may be several are) astray. We have, then, further to consider whether it is possible or convenient to attempt to rectify all these at once; and, if not, we have to decide which we should begin with.

Now this method has several recommendations. Amongst the first is, that it is not too high an ideal to be constantly aimed at. In a large number of cases it is closely allied to the principle which, as I have told you, many persons act upon—namely, the treatment of symptoms. Because every symptom of disease arises from the imperfect discharge of some function by its appropriate organ. Hence it requires only a slightly higher order of thought than that which is commonly in vogue. Indeed, many persons who profess only to treat symptoms, do rather aim at the treatment of functions. They misdescribe their principles, and so do themselves an injustice. Now, though this admission may indicate that the principle I am laying down is comparatively wanting in novelty, it is strong testimony to its practical utility. Practice would be much more imperfect than it is if all those who profess to treat symptoms really contented themselves with doing so. For this reason: if we treat a symptom merely, we often fail to remove that which causes the symptom. To give an extreme instance: If a man has a thorn in his hand, and we merely order opiates for the relief of the pain thus produced, we are very inadequately treating the disease, though we may be adequately treating the symptom. In some cases treatment based upon such a principle may actually aggravate the disease. You will remember that in my last lecture, which was upon certain urinary deposits, I pointed out to you that lithiuria sometimes produced impotency; and I mentioned to you that persons thus suffering, in whom the cause of the disorder had not been recognised, may, and are advised to, take tonics and live freely, both of which, and particularly the latter, have a direct tendency to aggravate the lithiuria, and so to increase the disorder they were intended to cure. Treatment of this kind is directed to the symptom—impotence—because in some such cases depending upon other causes, this treatment may be beneficial. If, then, every person who now professes to proceed upon the plan of treating symptoms would in each case profess to proceed upon the plan of treating functions, there would be a great gain to practical medicine. Miscarriages of treatment of the class I have pointed out—and they are very numerous indeed—would be avoided. Further, this principle would bridge over the lamentable chasm which now divides the science and art of medicine. Accomplished pathologists and morbid anatomists, whose true knowledge of disease has justly led them to recognise the incurability of many organic diseases, would enhance the benefits they have conferred on mankind if they would condescend to rectify those functions of which the apparatus is not totally disorganized, and thus much misery would be averted, and many lives eased and prolonged. On the other hand, those who find by experience the limited control which we can exercise even over functions, and the extreme difficulty with which they can in some instances be influenced, would gladly hail any assistance which science might afford them by its most recondite researches, when these throw any light upon the laws which govern the growth of cells, the distribution of the blood, or other mysterious acts upon which the discharge of function depends.

Another advantage is this : persons who got as far as I suggest would get further. It is impossible that any of you who have paid attention to cases in the hospital, and have watched or assisted in their investigation, can have failed to be struck by the fact that one and the same disorder of a particular function may in different cases arise from very different causes. Take, for example, epileptic fits. You have doubtless seen them, in the surgical wards, caused by external injury ; in the medical wards, you have seen them dependent upon the presence of worms in one case, upon disorder of the stomach in another case, and upon other causes in other cases. Now, knowing this, and resolving to treat not merely a symptom, which in the case of epilepsy is usually done by a miscellany of reputed specifics, you would naturally be led to examine into the case closely, and discover, if you could, the function which was permanently disordered, and the cure of which disorder (often lying very deeply hidden) would cure the more apparent and prominent phenomena, of which only the patient complains. Many cases of epilepsy may in this way be relieved. And remember this, that any specific must influence some function, though we may not know what function is disordered, still less the organ by which it is performed. In illustration of this, take the case of potassic iodide and secondary syphilis. A man has syphilis, and some time afterwards skin eruption, or sore throat, or falling of the hair, or painful swellings, or enlarged glands, or albuminuria, or anæmia, or iritis, or dry pleurisy, or simple cachexia, or a combination of these symptoms. We give him potassic iodide. The symptoms disappear, and there is no evidence that his health is not as perfect as in his pre-syphilitic period. Twelve months after, some of these symptoms return. What do we infer from this ? We assume that these symptoms arise from a poison in the system. If so, there must be some organ which manufactures this poison, and for a season ceased to do so; or else there must have been some organ which for a period ejected the poison from the system, and after a time ceased to do so; some organ, therefore, the peculiar function of which is deranged. What this organ is we know not—still less in what way its healthy action is modified. But the effect of the iodic salt has undoubtedly been to restore that function to its normal condition. So you see that Functional Medicine perfectly well admits of the employment of such a specific, though the discovery of its powers may have been purely accidental, and its *methodus medendi* be still unknown. Examples of the alteration of one function by the disorder of another might be multiplied almost infinitely. Take bronchitis only. Upon what an immense variety of causes, direct and indirect, may it not depend. Its treatment may or may not be influenced by our knowledge of its distant origin. It does not at all follow of necessity that the physician who treats it with simple expectorants, after, by minute inquiries, discovering many complications, is in error, though you may have thought so from my remarks in an early portion of this lecture. You see now that, in my opinion, Functional Medicine incorporates all the benefits of the imperfect method of symptom-treatment, and moreover leads directly on to the recognition and employment of the most precise, searching, minute, and scientific methods.

Another great recommendation of Functional Medicine, in my eyes, is, that it explains the necessity, and thereby justifies the use, of empirical plans and remedies. I will explain what I mean by empiricism. We seek to modify the function of the kidney. We have but a limited insight into the working of this organ; we know that it consists of certain parts, and we know to some extent the working of these parts. But we know very imperfectly. We find that in a state of health the quantity of the urinary secretion may be increased in numberless ways. When we come to use some of these means in a case of dropsy, where hyperuresis would be useful, they totally fail. We cannot tell why this is. Or we find that one amongst many succeeds. We can no more tell why this is. We only know the fact. Empiricism recognizes this imperfection of our knowledge; and it also tells us how best to counteract it. Do not persist in the use of a drug which does no good, or even possibly does harm, because, judging from some data or others, we have to come to the conclusion that it *ought* to do good; on the other hand, do not cast aside drugs or methods which seem to do good, or have done good, in apparently similar cases, because, judging from some data or others, they ought not to do good. Many proofs might be given that such a warning as this, superfluous as it may appear, is not really so. I will give but one. Dr. Bright, who was the chief discoverer of the diseases which go by his name, was in the habit of using diuretics in the treatment of them. I have always done so, and in many cases with signal success. Yet, in spite of this teaching by empiricism, their use has been strongly deprecated, because, judging by certain microscopical appearances, diuretics ought to be injurious. In treating a case of Bright's disease, I try to restore the function which is most seriously disordered. I find that in many cases, not in all, the only way to restore this function to something like its proper performance is to use diuretics.

Functional Medicine leads on a little further in another direction. It leads us to anticipate disease, and so to forestall the enemy. Thus we find that in the course of certain diseases there is special liability to interference with some particular function, as in the case of the kidneys in diptheria. Precautions may in this instance be readily taken which, though perhaps not infallibly, will probably prevent the functions of the kidneys being seriously interrupted, and thus we may prevent the very dangerous consequences which this interruption entail. We can similarly prevent scarlatinal dropsy.

Functional Medicine also justifies, and may therefore restore, some old and wise practices which have been submerged under the advancing wave of modern science. When it was not well recognized that many diseases tended naturally to cure themseves, methods were adopted for their relief which are now disused, not because they have been proved to be wrong in practice, but because they became unnecessary in theory. For example, the use of a sharp calomel purge at the outset of the exanthemata; once universal, is now rarely resorted to; yet the beneficial effects of this plan in ordinary cases need not be insisted on to those who in their own proper persons have been so treated. But where we find, from our knowledge of the natural history of a disease, that there is tendency to pernicious diarrhœa, as in typhoid, then we should either refrain from such treatment, or use it with extreme caution. This is another example of anticipation in Functional Medicine. Speaking of typhoid calls to my mind another example of the justification of an old treatment by Functional Medicine. I have never seen any more decided benefit produced by any treatment than I have seen, years ago in the Paris hospitals, from the application of leeches or cupping over the cæcum in certain cases of typhoid. Yet typhoid is a disease of debility, and we are urged nowadays in cases of debility to ignore all special functions and "restore" the patient. How, then, are we to explain the success of this anomalous treatment? Very simply. The function restored that part of the mucous membrane the disorder of which in the particular cases referred to, had aggravated the general disorder of the system. In the weakest patient it is often better to restore a function the irregularity of which is keeping the patient back, than to try to force them into strength by stuffing them with food and tonics and stimulants. How often do we see an enfeebled and disordered stomach thus overburdened and exasperated, instead of soothed by appropriate diet and quieting medicines.

The last consideration which I shall offer you as evidence of the propriety of the ideas which I have summed up in the term Functional Medicine may not be at first sight one of great practical weight. It is, nevertheless, in my estimation, anything but trivial. It is that this principle of action is in harmony with the latest and highest conceptions of philosophical medicine. To you who have probably not been much accustomed to abstract or metaphysical considerations, I may perhaps find a little difficulty in conveying my meaning. I will, however, try to do so in a plain and simple manner. What is disease? What do we mean by the term? In old times people used to think that a disease was some actual entity or thing which had got into the body some way, and was there lying hidden and secreted, and was to be cast out. We know that there are a few instances of this kind of thing. For example, we know that some disorders depend upon the presence of a tapeworm in the intestines, and that to cure them we must hunt the worm out of the body. This idea, which we now know to be true only in a few specific instances, was at one time general. The germ of this idea still runs through a great many of our conceptions of the nature of disease. This is more particularly the case in the pathological conceptions of ill-educated laymen. But many traces of it are still to be found amongst ourselves also. It is not long since the most illustrious and philosophical of existing pathologists has been dealing with this question; and the conclusion he has come to is that all diseases is disordered function. Here, then, I say, is the highest justification for all treatment being based upon the principle of restoring disordered functions to order, and this it is to which I have ventured to term Functional Medicine.—*Lancet, July* 1, 1871, *p* 4.

